# Clinical and immunological outcomes according to adherence to first-line HAART in a urban and rural cohort of HIV-infected patients in Burkina Faso, West Africa

**DOI:** 10.1186/1471-2334-14-153

**Published:** 2014-03-21

**Authors:** Emanuele Focà, Silvia Odolini, Giorgia Sulis, Stefano Calza, Virginio Pietra, Paola Rodari, Pier Francesco Giorgetti, Alice Noris, Paul Ouedraogo, Jacques Simpore, Salvatore Pignatelli, Francesco Castelli

**Affiliations:** 1 University Division of Infectious and Tropical Diseases, University of Brescia, Brescia, Italy; 2 Unit of Biostatistics and Biomatemathics, Department of Molecular and Translational Medicine, University of Brescia, Brescia, Italy; 3 Medicus Mundi Italy NGO, Brescia, Italy; 4 Saint Camille District Hospital, Nanoro, Burkina Faso; 5 Centre Medicale Saint Camille (CMSC), Ouagadougou, Burkina Faso; 6 Centre de Recherché Biomoleculaire Pietro Annigoni, Ouagadougou, Burkina Faso; 7 University Division of Infectious and Tropical Diseases, University of Brescia, School of Medicine, P.le Spedali Civili, 1-25123 Brescia, Italy

**Keywords:** HIV, Antiretroviral therapy, Adherence, Death, CD4+, Burkina Faso

## Abstract

**Background:**

Aim of our study is to investigate the clinical and immunological outcomes according to first-line HAART adherence in a large cohort of HIV-infected patients in Burkina Faso.

**Methods:**

A retrospective study was conducted between 2001 and 2009 among patients from two urban medical centers [St. Camille Medical Center (CMSC) and “Pietro Annigoni” Biomolecular Research Center (CERBA)] and 1 in the rural District of Nanoro (St. Camille District Hospital). Socio-demographical and clinical data were analyzed. Adherence was evaluated through a questionnaire investigating 5 key points related to drugs, consultations and blood exams, by assigning 0 to 2 points each up to 10 points overall. Data were collected at baseline and regularly thereafter. Adherence score was considered as a continuous variable and classified in optimal (8–10 points) and sub-optimal (0–7 points). Immunological outcome was evaluated as modification in CD4+ T-cell count over time, while predictors of death were explored by a univariate and multivariate Cox model considering adherence score as a time-varying covariate.

**Results:**

A total of 625 patients were included: 455 (72.8%) were females, the median age was 33.3 (IQR 10.2) years, 204 (32.6.%) were illiterates, the median CD4+ T-cell count was 149 (IQR 114) cells/μl at baseline. At the end of the observation period we recorded 60/625 deaths and 40 lost to follow-up. The analysis of immunological outcomes showed a significant variation in CD4+ T-cell count between M12 and M24 only for patients with optimal adherence (Δ=78.2, p<0.001), with a significant Δ between the two adherence groups at M24 (8–10 *vs* 0–7, Δ=53.8, p=0.004). Survival multivariate analysis revealed that covariates significantly related to death included being followed at CERBA (urban area) or Nanoro (rural area), and receiving a regimen not including fixed dose combinations, (p=0.024, p=0.001 and p<0.001 respectively); conversely, an increasing adherence score as well as an optimal adherence score were significantly related to survival (p<0.001).

**Conclusions:**

Adherence to HAART remains pivotal to build up a good therapeutic outcome. Our results confirm that, according to our adherence system evaluation, less adherent patients have a higher risk of death and of inadequate CD4+ count recovery.

## Background

It is estimated that about 32.2 million people were living with HIV at the end of 2012. Two thirds of HIV infections (approximately 23.5 million people) were in Sub-Saharan Africa, where a high prevalence of HIV/AIDS is reported (4.7%) [1]. The introduction of Highly Active Antiretroviral Therapy (HAART) in 1996 has considerably changed the clinical outcomes of infected patients, leading to a dramatic improvement in quality of life and decline in AIDS-related deaths in low-income Countries [2,3]. By the end of 2012, the number of people receiving antiretroviral therapy reached 9 million, meaning a 20-fold increase in treatment coverage compared to 2003 [1].

In many low-income Countries, where sometimes neither viral nor immunological parameters are available, adherence monitoring may be an acceptable instrument to estimate HAART effectiveness [4,5]. Besides, second-line regimens are still not easily available in these areas, thus making adherence to a first-line treatment even more important since its failure could leave the patients with no other therapeutic choices. Therefore, some studies have been carried on to better assess adherence patterns in Low and middle-income Countries [6,7] or to correlate adherence with patients outcome [8-11].

WHO usually defines adherence to any treatment as “the extent to which a person’s behavior-taking medication, following a diet, and/or executing lifestyle changes, corresponds with agreed recommendations from a healthcare provider”. Since no measurement strategy can be considered optimal especially when used alone, an holistic approach is currently suggested [12].

Actually, adherence assessment remains a rather challenging task: direct observation therapy is not practical for HAART regimens that have to be taken lifelong. Methods to establish adherence to antiretroviral treatment include pill count, pharmacy refill records, various self-reporting tools such as questionnaires and visual analogue scales, detection of drug blood levels and electronic monitoring devices [13]. The most commonly applied techniques in low-income Countries are pills count and self reports, though they often overestimate adherence [14].

On the other hand, it was demonstrated that the availability of various types of adherence support may avoid attrition to antiretroviral therapy [15].

Previously conducted studies concerning adherence to antiretrovirals found that a 95% minimum rate of drug assumption is required in order to guarantee a viro-immunological effectiveness of the therapy [16]. Also in Burkina Faso, the moderate spread of HIV drug resistance strains (from 5 to 15%) for both nucleoside reverse transcriptase inhibitors (NRTIs) and a non-nucleoside reverse transcriptase inhibitor (NNRTI) among HIV-infected women attending antenatal care, indicates that patients receiving antiretroviral therapy should be adequately supported in order to improve adherence and to avoid a further diffusion of resistance mutations [17].

Aims of this study are to explore death and CD4+ T-cell count evolution according to an original adherence score and to investigate predictors of death in a large cohort of HIV-infected patients over the first 24 months of treatment.

The study presented here was conducted within the European ESTHER (Ensemble pour une Solidarité Thèrapeutique Hopitalière en Reseau) project, a 9-year collaborative experience devoted to give assistance as well as technical and scientific support to fight HIV/AIDS in a low-income Countries.

## Methods

### Study design and population

This is a longitudinal observational single-cohort retrospective study that involved 3 different health facilities in Burkina Faso, 2 located in the suburban area of Ouagadougou [St. Camille Medical Center (CMSC) and “Pietro Annigoni” Biomolecular Research Center (CERBA)] and 1 in the rural District of Nanoro (St. Camille District Hospital). We enrolled all HIV-infected adults who started HAART according to WHO international guidelines available in the study period [18,19], between January 2001 and February 2009. Patients were followed since HAART beginning for at least one year afterwards, two years when feasible.

All data were collected in Burkina Faso (a West-African country with a population of about 13.2 million people) which is among the poorest countries in the world (181st position out of 187) [20]. Notably, in this country only 49% of the 64000 estimated HAART-needing patients were actually receiving it by the end of 2010 [17].

### Data collection

Consultations and data collection were performed by a medical doctor, available for both scheduled and emergency visits. Routine clinical visits where conducted according to WHO international guidelines [18,19]. Patients enrolled in the study, were evaluated at baseline (D_0_), on day 15 (D_15_), each month during the first semester (M_1_-M_6_) and thereafter every three months since HAART initiation. Each time, WHO clinical stage and adherence score (from D_15_ on) were registered. Furthermore, CD4+ T cell count was scheduled on D_0_ and then at M_6_, M_12_ and M_24_, together with some routine laboratory tests (i.e. blood cell count, transaminases, creatinine, amylasemia and glycaemia). During the study, patients were categorized into (i) followed, (ii) lost to follow-up, (iii) dead and (iv) referred to other Medical Centers. A patient was considered lost to follow up if he/she had missed 2 consecutive visits without a further contact. Demographical and clinical variables, social behaviors, family conditions and literacy were also analyzed.

### Adherence score

Adherence was assessed through the administration of a questionnaire regarding 5 different aspects: (1) pills count, (2) delays and omissions in taking pills referred from patient, (3) knowledge of posology, (4) punctuality on consultations, (5) proper execution of blood tests as planned at the previous consultation. A score was estimated by the medical doctor who performed the visit through the assignment of 0 (i.e. unreached goal), 1 (i.e. partially reached goal) or 2 (i.e. fully reached goal) points to each feature, up to 10 points overall. This score has not been validated. Treatment interruption was determined as a referred and/or planned history of stopping and resuming treatment, and corresponds to a zero score. On the other hand, a full adherence score was attributed to patients (1) who took at least 95% of pills (as stated through direct count and self report of missed doses), (2) at the right time of the day (with 30 minutes tolerance), (3) who properly knew the regimen posology, (4) presented on scheduled consultations (± 2 days, since extra-medications were provided) and (5) underwent blood tests as planned. If a patient was lost to follow up (see above), no value was assigned to the adherence score, and the patient was considered in the statistical analysis only according to the period of follow-up.

### Statistical analysis

Associations between categorical variables were evaluated using Chi-square tests or Fisher exact tests where needed. Overall survival was modeled using Cox models considering adherence score as time-varying covariate. The following variables were considered in univariate analyses: center, age (continuous), gender, religion, living area, HIV type infection, literacy, CD4+ T-cell count, WHO clinical stage, HAART regimen including fixed dose combination (FDC), calendar year and adherence score (as continuous and categorical [0–7,8–10] variable) and those significant were included in a multivariate Cox model. Model selection for the multivariate Cox model was based on likelihood ratio tests. The final model included center, HAART regimen including FDC, calendar year, WHO clinical stage and adherence score (as continuous and categorical [0–7,8–10] variable). Both calendar year and WHO clinical stage were included as a stratification variable. When performing the survival analysis, all patients lost to follow-up were censored.

CD4+ T-cell count variation over time was modeled using a random-intercept linear mixed model [21] with CD4+ T-cell expressed on the squared root scale. Model selection was performed based on likelihood ratio tests both for fixed and random effects of the model. Within-subjects errors were auto-correlation was modeled using a continuous first-order autoregressive process (CAR1) with an estimated autocorrelation coefficient of 0.66. The final model included sex, age (continuous), center, WHO HIV clinical stage, HAART regimen including FDC, baseline CD4+ T-cell count, adherence score (categorized as [0–7,8–10]), HIV type infection (HIV-1, HIV-2 or HIV 1 and 2 mixed) as well as HAART regimen including FDC-time and score-time interaction terms.

Considering the possibility of a missing not at random pattern and its potential effects on model estimates we conducted a sensitivity analysis fitting a linear mixed model as specified above only on subjects that had a visit at 24 months. Results did not differ from the model with all data (data not shown).

All analysis were performed using the R statistical software [22] and specifically the packages *survival*[23] and *nlme*[24]. All statistical tests were performed based on a significance level of 5%.

### Ethics

The Study was conducted in compliance with the Helsinki declaration. The Joint Centre Médicale Saint Camille and Centre de Recherche Biomoléculaire Pietro Annigoni Ethics Committee were duly informed about the ongoing research and gave their permission. In fact, the CNERS (Comité National d’éthique pour la Recherche en Santé) is questioned only in case of interventional clinical trials as well as in international or national pharmacological studies: therefore in this case it was not informed. Moreover, being a retrospective non-interventional study founded on post hoc analysis of data already present in patients’ files, and collected only for clinical indications, no written informed consent was asked to patients.

## Results

### Patients characteristics

A total of 625 HIV-infected patients who started HAART between January 2001 and February 2009, were included. Socio-demographic and clinical features of this cohort are showed in Tables 1 and 2 respectively. Patients were followed for a median time of 20.8 months (min. 0.6 – max 24 months). Most individuals were female (455/625, 72.8%) and the median age at enrollment was 33.3 (IQR 10.2) years. Moreover, a large proportion of patients had a stable partner (59.3%), many were widowed (23.0%) and 17.1% had no stable partner. With respect to literacy, 32.6% (204/625) of patients were illiterate. Notably, only 57.8% of enrolled subjects spoke French, nearly all the others knew local dialects only. The 74.6% (466/625) of patients were living in Ouagadougou, while the remaining 25.4% (159/625) were from rural areas.

**Table 1:** Socio-demographic characteristics of patients at baseline

**Variable**	**All patients (n = 625)**
**Sex**	
-Female	455/625 (72.8)
-Male	160/625 (27.2)
**Median age at baseline**	
Years [median (IQR)]	33.3 (10.2)
**Religion**	
-Christian	368/625 (58.9)
-Muslim	275/625 (40.8)
-Animist	2/625 (0.3)
**Spoken language**	
-French	361/625 (57.8)
-Only local dialects	237/625 (37.9)
-Missing data	27/625 (4.3)
**Literacy**	
-Illiterate	204/625 (32.6)
-Literate	421/625 (67.4)
**Living area**	
-Urban	466/625 (74.6)
-Rural	159/625 (25.4)
**Calendar year of enrolment in the whole cohort**	
-≤ 2003	71/625 (11.4)
-2004	166/625 (26.6)
-2005	130/625 (20.8)
-2006	78/625 (12.5)
-2007	111/625 (17.8)
-2008	69/625 (11)
**Marital status**	
-Stable partner	371/625 (59.3)
-No stable partner	107/625 (17.1)
-Widowed	144/625 (23.0)
-Missing data	3/625 (0.5)
**Pregnancy at baseline**	
-Yes	73/455 (16.1)
-No	381/455 (83.7)
-Missing data	1/455 (0.2)
**HIV-infected partner**	
-Yes	199/408 (48.8)
-No	82/408 (20.1)
-Missing data	127/408 (31.1)
**Status at the end of follow-up**	
-Followed	488/625 (78.1)
-Lost to follow-up	40/625 (6.4)
-Dead	60/625 (9.6)
-Referred	37/625 (5.9)

Notes: IQR: Interquartile range. All values are expressed as N (%).

**Table 2:** Clinical characteristics of patients at baseline

**Variable**	**All patients (N = 625)**
**Median CD4+ T cell count at baseline** (cells/μl)	
cells/μl [median (IQR)]	149 (114)
*[calculated on 593/625 pts]*	
**CD4 T cell count at baseline**	
-< 50 cells/μl	88/593 (14.8)
-> 50 cells/μl	505/593 (85.2)
**HIV-type infection**	
-HIV-1	587/625 (93.9)
-HIV-2	10/625 (1.6)
-HIV-1 + HIV-2	28/625 (4.5)
**WHO clinical stage at baseline**	
-Early (stage 1 and 2)	309/625 (49.4)
-Advanced (3 and 4)	316/625 (50.6)
**HAART regimen**	
-2NRTIs + 1NNRTI	549/625 (87.8)
-2NRTIs + 1PI/r	76/625 (12.2)
**HAART regimen including FDC**	
-Yes	474/625 (75.8)
-No	151/625 (24.2)

Notes: IQR: Interquartile range; HAART: highly active antiretroviral therapy; NRTIs: nucleoside reverse transcriptase inhibitors; NNRTIs: non nucleoside reverse transcriptase inhibitors; PI/r: protease inhibitors boostered by ritonavir; FDC: fixed dose combination; All values are expressed as N (%).

At the beginning of antiretroviral treatment, a significant proportion of patients belonged to an advanced WHO clinical stage (50.6%, 316/625) and the median CD4+ absolute count was 149 cells/μl (IQR 114). Overall, 63/625 patients (10.1%) had a concurrent HBV or HCV chronic infection. The 87.8% (549/625) of prescribed HAART regimens consisted of two nucleoside reverse transcriptase inhibitors (NRTIs) and a non-nucleoside reverse transcriptase inhibitor (NNRTI), while the remaining 12.2% (76/625) were made up of two NRTIs and a protease inhibitor (PI). Moreover, 75.8% (474/625) of patients received FDC (e.g. Zidovudine/Lamivudine, Stavudine/Lamivudine/Nevirapine, Stavudine/Lamivudine).

By the end of follow-up, 9.6% of patients (60/625) had died, 5.9% (37/625) had been referred, 6.4% (40/625) were lost to follow-up and 78.1% (487/625) were still under active monitoring. Median follow-up for subject censored before the end of the study (24 months) was 8.97 months, in particular median follow-up for dead patients was 6.54 months.

### CD4+ T cell count increase according to adherence score

**Figure 1:**
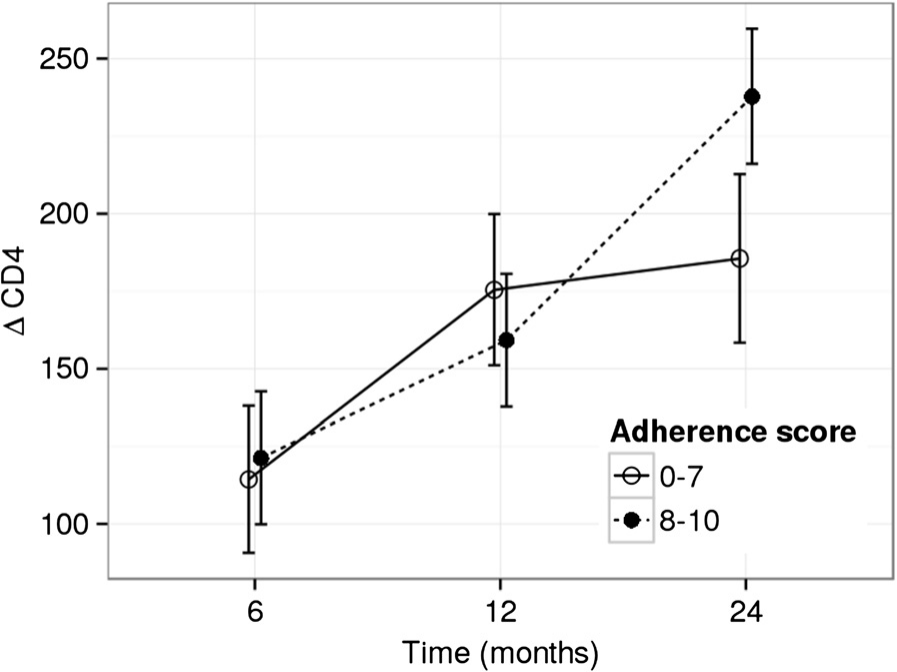
**Estimated mean Δ CD4+ T cell count evolution.** The plot represents estimated mean Δ CD + T counts separately for optimal (8–10) and suboptimal (0–7) adherence levels at every visit (6 months, 12 months and 24 months). Bars represent the standard error of the means.

To evaluate the immunological outcome, we considered two different groups of patients based on adherence score considered at each time-point. We divided patients with optimal adherence (8–10 points) from those with sub-optimal adherence (0–7 points). We observed a significant increase in CD4+ T-cell count from baseline to M6, both in the 0–7 (Δ = 112.4, p < 0.001) and 8–10 groups (Δ = 120.7, p < 0.001), while the difference between the two groups at M6 was not significant (p = 0.56). Moreover, from M6 to M12 we found a CD4+ T-cell count significant variation in both adherence groups (Δ = 62.6 p < 0.001 and Δ = 38.1 p < 0.001 in the 0–7 and 8–10 group respectively), and a non-significant difference was observed between the two groups at M12 (p = 0.39). A significant variation was observed between M12 and M24 only in the 8–10 group (Δ = 78.2, p < 0.001) with a significant Δ between the two groups at M24 (8–10 *vs* 0–7, Δ = 53.8, p = 0.004). Figure 1 shows the estimated Δ (from G0) and their standard errors.

### Survival and predictors

Univariate and multivariate logistic regression analysis were conducted to explore the possible predictors of death as summarized in Table 3. In univariate analysis factor independently associated with death were: being followed at CERBA or Nanoro (p <0.001 for both variables), living in rural area, increasing age, early HIV disease clinical stage and receiving a HAART regimen not including FDCs (p = 0.008, p = 0.076, p = 0.013 and p < 0.001 respectively). Besides, factors inversely related to death were increasing adherence score (for 1-point increase) and optimal adherence score (8–10 point), both considered as time-varying covariates (p < 0.001 and p < 0.001 respectively). In the multivariate model covariates significantly associated with death included being followed at CERBA or Nanoro and receiving a regimen not including FDCs, (p = 0.024, p = 0.001 and p < 0.001 respectively); the increasing adherence score and the optimal adherence score (8–10 points) were confirmed to be significantly related to survival (p < 0.001 for both).

**Table 3:** Predictors of death: univariate and multivariate analysis

		**Univariate analysis**			**Multivariate analysis**		
	**Variables**	**HR**	**95% IC**	**p value**	**HR**	**95% IC**	**p value**
Center	CERBA vs CMSC	4.98	2.19 – 11.32	**<0.001**	2.76	1.15 – 6.65	**0.024**
	Nanoro vs CMSC	8.08	3.35 – 19.49	**<0.001**	10.60	3.78 – 29.75	**0.001**
Sex	M vs F	1.44	0.83 – 2.51	0.21			
Religion	Muslim vs Christian	0.90	0.52 – 1.54	0.69			
Living area	Rural vs Urban.	2.12	1.24 – 3.64	**0.008**	-	-	-
HIV type infection	2 vs 1	1.29	0.18 – 9.38	0.80			
	Mixed vs 1	2.31	0.92 – 5.78	0.075			
Literacy	Illiterate vs literate	1.29	0.75 – 2.23	0.36			
Age	1 year increase	1.32	0.98 – 1.79	0.076			
CD4+ T-cell count at baseline	≤50 vs >50	1.53	0.74 – 3.17	0.28			
WHO HIV clinical stage^$^	Early vs advanced	0.5	0.29 – 0.88	**0.016**	-	-	-
HAART regimen including FDC	No vs Yes	2.70	1.58 – 4.59	**< 0.001**	0.40	0.22 – 0.70	**<0.001**
Increasing adherence score	For 1 point increasing	0.60	0.47 – 0.76	**<0.001**	0.87	0.81 – 0.93	**<0.001**
	8–10 vs 0–7	0.37	0.21 – 0.63	**<0.001**	0.32	0.18 – 0.57	**<0.001**
Calendar year*^$^	-	-	-	**0.008**	-	-	-

Notes: HR: hazard ratio; CI: confidence interval; CERBA: “Pietro Annigoni” Biomolecular Research Center; CMSC: Centre Médicale Saint Camille, Ougadougou; HAART: highly active antiretroviral therapy; FDC: fixed dose combination.

*For Calendar Year only p-value for likelihood-ratio test (model with/without variable) reported.

^$^Variables included in the model as strata.

^§^p-values below 0.05 (in boldface) were considered to be significant.

## Discussion

In this study we observed a direct correlation between the two different outcomes (survival and CD4+ T-cell count recovery) and adherence evaluated through our score: our data substantially confirm previous findings [25,26] suggesting that good adherence enhances CD4+ T-cell recovery and decreases mortality.

A significant proportion of our study population was represented by women (72.8%), since CMSC is a medical center devoted to “mother and child health-care” where patients are predominantly pregnant women and their HIV-infected partners [27]. Hence, patients who went to CMSC were usually asymptomatic at enrolment (i.e. at an early WHO clinical stage at baseline). Conversely, the other two centers mainly admit symptomatic individuals and hospitalized late presenters.

Moreover, people coming from Nanoro rural District often emigrate particularly to Ivory Coast, delaying access to HIV screening and care [28]. The high proportion of HIV-infected migrants on HAART who work abroad may be partly responsible for the worsening of therapeutic adherence in this setting. This phenomenon was taken into account during the statistical analysis and the multivariate logistic regression model was realized after stratifying for WHO clinical stage at baseline and for calendar year.

The lack of a gold standard method to evaluate adherence brought us to use a multi-parametric strategy based on a combination of questionnaires, pills count, punctuality on clinical consultations and blood tests. This method allowed us to bypass single measurements–related biases, by taking into account several aspects of treatment adherence according to WHO recommendations [12]. Previous studies employed a variety of methods to assess adherence, with different thresholds to define good adherence, and a wide range of adherent patients proportions were reported (from 20.7 to 93.5%) [10,11,29-34]. Since the methodologies of these studies widely varied from each other, they are not easily comparable to our research.

The increase in CD4+ T-cell count was similar in patients with optimal and suboptimal adherence (0–7 and 8–10 group) during the first year of follow-up, while a better adherence (8–10 group) during the second year was significantly related to a progressive increase in CD4+ T-cell count. Our findings are in line with those described in the DART trial where investigators observed a worse immunological recovery (assessed through the CD4+ T-cell count evolution) among less-adherent patients.

We also found that good adherence is strongly related to a lower risk of death in univariate and multivariate models, given that the adherence score was considered as continuous variable or as a binomial variable (8–10 *vs* 0–7 points).

The univariate analysis of death predictors revealed that living in urban areas was associated to a better survival, while the other socio-demographic features (education, religion, gender and increasing age) were not. In fact, living in a urban area is likely to be associated with fewer disruptions in access to medicines, which seem to facilitate adherence [35-37]. Moreover, WHO clinical stage at baseline resulted to be significantly related to death, while CD4+ T-cell count (<50 *vs* >50 cells/μl) did not.

The multivariate analysis showed an increased risk of death among patients who were followed at CERBA or Nanoro as compared to those followed at CMSC, as a consequence of the above-mentioned differences between patients enrolled in the three healthcare facilities.

In addition, receiving a regimen including FDCs was confirmed to be significantly related to survival, suggesting the importance of a wider access to HAART in low-income Countries with a particular attention to compact regimens [38].

At any rate, some limitations should b e taken into consideration while interpreting our study. First of all, it was not possible to collect full data for the whole cohort since medical records were sometimes incomplete or because patients had died, had been lost to follow-up or transferred elsewhere. Second, our adherence score has not been validated and we are not able to establish the relative weight of each component of the score. Moreover, we did not compare this score with other methods to evaluate adherence since validation of this score was not the aim of this study. Lastly, the assignation of our score is purely at the discretion of the physician who performed the visit, and it should not be interpreted as a objective measure. Although the scores assigned at each visit were equally weighted, despite the variability in the length of assessment, the patients were followed in accordance with current WHO guidelines and all data were analyzed only retrospectively.

## Conclusions

In summary, our findings confirm that optimal adherence is strongly related to better immunological recovery and survival, though assessed through a non-validated score. Our results reinforce the need of additional support such as reminders and enhanced community education [38]. In fact, HIV/AIDS – related stigma still represents a major concern in low-income Countries especially in Sub-Saharan Africa and it is thought to significantly influence patients’ compliance. Notably, scaling up antiretroviral treatment coverage alone is not sufficient: adherence improvement remains necessary, with a particular attention to prescribe FDC-containing regimens in order to achieve both clinical and immunological outcomes.

## Competing interests

EF have received travel grants or speakers honoraria from several Pharmaceuticals Companies producing antiretroviral drugs and consultancy fees from Gilead and Abbvie but this did not influence the content of this paper. FC is acting as Principal Investigator in investigational Clinical Trials sponsored by Pharmaceuticals Companies producing antiretroviral drugs. The remaining authors declare that they have no competing interests.

## Authors’ contributions

Study concept and design: EF, SO, VP. Acquisition of data: EF, SO, AN. Analysis and interpretation of data: EF, SO, GS, SC, VP. Drafting of the manuscript: EF, SO, GS, PFG. Statistical Analysis: SC. Critical revision of the manuscript for important intellectual content: EF, SO, GS, SC, VP, PR, PFG, AN, PO, JS, SP, FC. All authors read and approved the final manuscript.

## Pre-publication history

The pre-publication history for this paper can be accessed here:

http://www.biomedcentral.com/1471-2334/14/153/prepub
